# Evaluating the effectiveness and cost-effectiveness of a digital, app-based intervention for depression (VMood) in community-based settings in Vietnam: Protocol for a stepped-wedge randomized controlled trial

**DOI:** 10.1371/journal.pone.0290328

**Published:** 2023-09-05

**Authors:** Leena W. Chau, Jill K. Murphy, Vu Cong Nguyen, Hui Xie, Raymond W. Lam, Harry Minas, Yufei Zheng, Emanuel Krebs, Kanna Hayashi, Son Dao, Xuan Nguyen, Viet Anh Duong, Eugene Fiume, John O’Neil

**Affiliations:** 1 Faculty of Health Sciences, Simon Fraser University, Vancouver, Canada; 2 Faculty of Medicine, Department of Psychiatry, The University of British Columbia, Vancouver, Canada; 3 Institute of Population, Health and Development, Hanoi, Vietnam; 4 Global and Cultural Mental Health Unit, Centre for Mental Health, Melbourne School of Population and Global Health, University of Melbourne, Melbourne, Australia; 5 Faculty of Applied Sciences, Simon Fraser University, Vancouver, Canada; PLoS ONE, UNITED STATES

## Abstract

The COVID-19 pandemic has amplified mental health problems and highlighted inequitable gaps in care worldwide. In response there has been an explosion of digital interventions such as smartphone applications (“apps”) to extend care. The objective of this trial is to evaluate the effectiveness and cost-effectiveness of a digital depression intervention (*VMood*), delivered via a smartphone app. VMood is adapted from an in-person intervention that was delivered by non-specialist providers and shown to be effective in the Vietnamese context in our previous trial (2016–2019). A stepped-wedge, randomized controlled trial will be conducted across eight provinces in Vietnam. Adults aged 18 years and over will be recruited through community-based primary care centres and screened for depression using the embedded Patient Health Questionnaire-9 (primary outcome measure). Participants scoring 10–19, indicating depression caseness, will be randomly allocated to the intervention or control group until the target of 336 is reached. Secondary outcome measures will examine the effect of the intervention on commonly co-occuring anxiety, quality of life and work productivity, along with use of alcohol and tobacco products. Assessments will be administered through an online survey platform (REDCap) at baseline, and at every 3 months until 3 months post-intervention. Intervention-group participants will receive VMood for a 3-month period, with online support provided by social workers. Control-group participants will receive a limited version of the app until they cross into the intervention group. Generalized Linear Mixed-effect Models for clustered measures will be used for all outcomes data. We will conduct a cost-effectiveness analysis alongside the trial to capture VMood’s costs and benefits. This trial will provide evidence on the effectiveness and cost-effectiveness of a digital mental health intervention adapted from an in-person intervention. This trial will also contribute important information to the growing and promising field of digital mental health.

**Trail regulation.** Registered at ClinicalTrials.gov, identifier [NCT05783531].

## Introduction

### Background and rationale

Mental disorders account for 13% of the global burden of disease [1]. Global prevalence rates for depression and anxiety disorders, the two most common mental disorders [2], are estimated at 4.4% and 3.6%, respectively [3], with anxiety frequently co-occurring with depression [4]. Despite this, there is an overwhelming gap in mental health treatment, especially in low- and middle-income countries (LMICs) [5], which bear 80% of mental disorders disease burden [6]. A major contributor to the treatment gap is a shortage of health human resources (e.g., psychiatrist, psychiatric nurses) [7]. In Vietnam, an LMIC, the treatment gap for depression is 83% [8]. The very limited human resources for mental health are generally available only in tertiary-care facilities in large urban centres [9], where depression care is focused predominantly on pharmacological treatment in the critical absence of counseling and psychosocial interventions [9]. Epidemiological evidence suggests that depression prevalence in Vietnam is similar to global rates [10,11]. Most of the estimated four million persons living with depression in Vietnam, particularly those living in the country’s rural and remote areas, have very limited access to treatment, care and support.

The COVID-19 pandemic has had a profound impact on population mental health, with certain populations, such as those residing in rural and remote areas with limited access to resources, disproportionately affected [12]. The pandemic has also amplified existing gaps and inequities in mental health care [13–15], leading to an increased urgency to expand crucial evidence-based and low-barrier mental health care. The severe and persistent shortage of human resources for mental health is a continuing impediment to meeting population mental health service needs. The necessary shift to remote care delivery during the COVID-19 pandemic lockdowns has highlighted the promise of digital health interventions, including smartphone apps, as a feasible means to scaling up access to mental health services [16,17]. In response there has been an explosion of smartphone apps for common forms of mental illnesses, including depression and anxiety, as many countries recognize the opportunity for harnessing the capabilities of digital technologies globally. Current evidence for these interventions, however, is limited [17–19].

The annual economic costs directly attributable to mental illnesses is estimated at USD $1.15 trillion globally [8]. The returns on investment for scaling-up the response to the massive public health and economic burden of depression, therefore, have been suggested to be substantial, with an estimated benefit to cost ratio of 5.7 in LMICs when the value of health and economic benefits is considered [8]. A recent systematic review on economic evaluations of digital health interventions for depression has provided promising evidence for the cost-effectiveness of such interventions [20]. However, all studies included in the review were from high-income countries, further underlining the large gap in the evidence-base on the cost-effectiveness of interventions for depression in LMICs [21].

The objective of this trial is to test the effectiveness and cost-effectiveness of a digital mental health intervention called VMood in Vietnam. VMood is a smartphone-based depression intervention adapted from an in-person Supported Self-Management (SSM) intervention developed in Canada. SSM is a low-cost approach where providers support people to recover from depression [22]. It is grounded in principles of Cognitive Behavioural Therapy and utilizes an *Antidepressant Skills Workbook* (ASW) and a task-sharing approach, wherein non-specialist providers (e.g., lay health worker, community health worker) are trained to provide coaching support to individuals working through the ASW [22]. Task-sharing has emerged as a key strategy for scaling up mental health services, especially in low-resource contexts [2,23,24].

From 2016–2019, we conducted a trial to test the effectiveness of the in-person SSM intervention in community-based settings across eight provinces in Vietnam [25]. Non-specialist providers, supervised by primary care providers, delivered support to 376 adults with depression caseness (defined as score of ≥8 on the Self-Reporting Questionnaire [SRQ-9]) across 8 provinces in Vietnam. Two districts and two communes (municipal subdivisions) were randomly selected from each province, for a total of 16 districts and 32 communes. Randomization occurred at the commune level. The primary outcome was the effect of the SSM intervention on change in depression scores, and the primary analysis was intention-to-treat. Results showed that receiving the SSM intervention reduced the odds of having depression by 58% compared with the control group, who received care as usual, demonstrating that accessible treatment by non-specialists in community settings is effective for mild to moderate depression [25].

In Vietnam, the Ministry of Labour, Invalids, and Social Affairs (MOLISA) and Ministry of Health (MOH) are jointly responsible for managing mental health services, with MOH responsible for the national psychiatric hospitals and MOLISA responsible for the Social Protection Centres that provide rehabilitation and social support services [26]. Over the past two and a half decades, the Government of Vietnam has focused increasing attention on strengthening its mental health system, implementing national programs of reform aimed at improving population mental health [26], culminating in the recent national program on Social Assistance and Functional Recovery for People with Disabilities, Autistic Children, and People with Mental Disorders in the 2021–2030 period, a commitment to community-based programs and information technology for mental health promotion and management [27]. This is in recognition of the key role digital health interventions can play in the context of severely constrained resources for mental health that are likely to persist [28] and the pressing need for virtual care amidst and beyond the COVID-19 pandemic.

In response, we adapted the in-person SSM intervention to a digital intervention (*VMood*), to be delivered via a smartphone app. VMood consists of three-months’ engagement with the VMood program components, adapted from the ASW, and online coaching support delivered by a MOLISA social worker virtually through the app. This paper describes the trial protocol for this study that will assess the effectiveness of the VMood intervention, beginning with feasibility testing. Alongside the trial a cost-effectiveness analysis will be conducted to capture the costs and benefits of the intervention. With ongoing support from the Government of Vietnam, we will implement VMood across eight provinces in Vietnam.

## Trial design

### Study objectives

The purpose of this study is to test the effectiveness and cost-effectiveness of a digital, app-based supported self-management (SSM) intervention for depression, adapted from a previously-tested [25] in-person SSM intervention. Specifically, we hypothesize that the digital intervention (VMood, which includes modified SSM) is superior to a control condition (the app delivering health information only) for reducing depression caseness among Vietnamese community-based adult populations screening positive for depression, and that the use of VMood will be cost-effective.

### Overall study design

The study will use a cluster stepped-wedge randomized controlled trial (RCT) design, with three stepped-wedges. Cluster stepped-wedge designs are pragmatic and appropriate for balancing scientific rigour with real world conditions, including the logistic and time constraints often faced by policy makers and health systems [29]. Stepped-wedge designs also represent an ethical approach in circumstances where access to evidence-based interventions is limited because they allow all participants to access the intervention as all clusters cross from the control into the intervention group [29].

In this study, randomization will occur at the commune level and communes will be assigned to receive either the VMood intervention or the control condition first. Participants in communes assigned to the control condition will be provided access to the VMood app and screening and assessment measures, but content will be limited to a video presenting general information about depression. In a typical stepped-wedge design, when a cluster crosses over from control to intervention, they remain in the intervention arm until the study is complete. In this case the intervention period will last three months in accordance with the VMood protocol. We will also collect follow-up data from each cluster three months after they have completed the intervention. Table 1 illustrates the timing of the intervention and control groups in the stepped-wedge design for one study district. Due to the size of the study, which will include 8 districts and 48 communes in total, all communes in the same district will begin the study at the same time but the entry of provinces will be staggered. The study has not started recruiting participants.

**Table 1:** VMood stepped wedge design by month—one district.

		1	2	3	4	5	6	7	8	9	10	11	12									
C1																						
C2																						
C3																						
C4																						
C5																						
C6																						
		Baseline																				
		Intervention																				
		Control																				
		Follow-up																				
	C	Commune																				

### Participants

#### Setting

The study will be carried out in eight Vietnamese provinces that represent diversity in terms of population composition, economic status and availability of mental health service resources. The study will take place in one district of each province, including six communes (municipal subdivisions) in each district for a total of 48 communes [see Table 2]. For logistical reasons, selected provinces represent a mix of provinces that previously participated in the in-person RCT and those that have not, though different districts and communes will be selected in all provinces that participated in the previous study to avoid bias.

**Table 2:** Participating provinces, districts, and communes.

Province	District	Communes
1	1	1, 2, 3, 4, 5, 6
2	2	1, 2, 3, 4, 5, 6
3	3	1, 2, 3, 4, 5, 6
4	4	1, 2, 3, 4, 5, 6
5	5	1, 2, 3, 4, 5, 6
6	6	1, 2, 3, 4, 5, 6
7	7	1, 2, 3, 4, 5, 6
8	8	1, 2, 3, 4, 5, 6

#### Inclusion and exclusion criteria

The trial will be implemented so that participants enter the study in a naturalistic manner. Community meetings will be organized to promote VMood, and all commune members will be invited to download the app, complete registration and informed consent and then complete the nine-item depression screening measure (Patient Health Questionnaire; PHQ-9 [30]). Healthcare staff members, funded by MOH, at the commune health stations and outpatient clinics will assist with publicizing the VMood app and encourage commune members to download it. Healthcare workers will be provided with information about the intervention as well as about the informed consent required to participate.

Inclusion criteria for the study are: 1) participant meeting depression caseness criteria based on the PHQ-9 measure (PHQ-9 score between 10–19) and 2) informed consent to participate in the study and complete all outcomes measures. Exclusion criteria include participants experiencing severe mental illness (PHQ-9 ≥ 20), including severe depression, suicidal ideation, or symptoms of other serious mental health conditions, who will be referred via an in-app notification to psychiatric and/or emergency care.

#### Informed consent

At registration, after downloading the VMood app, participants will be required to complete an online written informed consent form via the app in order to proceed to the contents of the app. All consent information collected through the app will be stored digitally. Participants needing support to complete the consent form or who have any questions will be provided with contact information for a member of the study team, who they can contact to complete the consent form either verbally (by phone) or by email (signed electronic copy).

### Interventions

#### Intervention condition

Supported self-management (SSM) is an evidence-based approach to the management of chronic disease which empowers the patient to participate in their own care by building appropriate skills with the support of a physician, lay provider, or other carer [31]. The in-person SSM for depression intervention that was previously tested [25] included a cognitive-behavioural therapy (CBT)-informed bibliotherapy intervention provided via the ASW [32] with supportive coaching provided face-to-face by non-specialist providers [33]. For the digital intervention used in this study, ASW was adapted to be delivered via the VMood smartphone app, with supportive coaching as needed provided through an online chat function by social workers. The VMood app maintains the core, skills-based components of the ASW, which include skill-building modules on behavioural activation, problem-solving, and realistic thinking. The ASW content will be delivered using multiple modalities, including text, video and audio recordings explaining the core concepts, and activities including quizzes and exercises that help participants to apply the skills to their own lives. Users will also be able to track their symptoms and monitor improvement with repeated PHQ-9/GAD-7 assessments at monthly intervals. VMood will also include features such as reminders and in-app notifications to promote engagement. As described above, the process of adapting the SSM intervention for app-based delivery and fidelity and usability testing will be reported elsewhere.

#### Control condition

The control condition will be enhanced treatment as usual. Depression is not widely recognized or treated in primary care in Vietnam and it is therefore likely that participants who meet the caseness criterion for depression based on PHQ-9 score will receive little or no treatment for depression. Enhanced treatment as usual will consist of access to the VMood app that has only the Introductory Modules turned on. These modules include the in-app screening measure (PHQ-9, with ongoing tracking) and the introductory video about depression, which describes symptoms and features of depression. Participants in the control condition will be informed that they are queuing for the intervention until they receive the intervention and, as described above, will receive access to the VMood introductory modules that provide information about depression and to the in-app screening measure (PHQ-9). The control VMood app will also include immediate referral to tertiary and/or emergency care for participants identified as having severe depression at screening (PHQ-9 ≥ 20) or who indicate they are experiencing suicidal ideation based on endorsing Question 9 on the PHQ-9. Participants in communes randomized to the control condition will be offered access to VMood’s full features, including the app-based SSM, following the period of control data collection as illustrated in Table 1.

### Outcomes

#### Primary outcome

The primary outcome of the study is depression caseness (PHQ-9 score ≥10 based on the Vietnamese version of the PHQ-9 [30] assessed at 3- and 6-months after initiation of the VMood intervention. The PHQ-9 and has been identified as the most suitable measures for use in a mobile app [16] and have been validated for use in Vietnam [30]. We will also collect demographic measures, including: age, sex, gender (if different from assigned sex at birth), education level, marital status, ethnicity, migrant status, employment status and classification, social insurance status and prior hospitalization due to mental illness.

#### Secondary outcomes

Secondary outcome measures will contribute to study findings across several categories, including clinical, quality of life, economic, and usability. Other clinical outcomes include changes in PHQ-9 and GAD-7 score from baseline to post-intervention. The effect of the intervention on quality of life, including dimensions such as social relationships, perceived well-being, and overall health [34], will be measured using the WHO Quality of Life- Brief (WHOQOL-BREF) [35]. Changes in use of alcohol and tobacco products during intervention use will be measured using the Fast Alcohol Screening Test (FAST) [36] and the Alcohol, Smoking and Substance Involvement Screening Test (ASSIST) [37] adapted to assess tobacco use only. We will also collect data on any other care for depression that participants in both the intervention and control group receive during the course of the study from the HEA.

For the cost-effectiveness analysis (details are presented below), we will estimate health-related quality of life using the EQ-5D-5L [38]. The EQ-5D-5L instrument is available in Vietnamese and has been validated in Vietnam, with country-specific weights based on social preferences obtained from a nationally representative sample available for the calculation of QALYs [39]. Costs attributable to health resource utilization will be estimated from self-reported inpatient and outpatient visits using the Health Economic Assessment (HEA) [40] adapted for the Vietnamese health system context. Indirect costs attributable to potential productivity gains will be measured using the validated Work Productivity and Activity Impairment Questionnaire: Depression (WPAID:D) [41,42].

Finally, the System Usability Scale (SUS) [43] will be used to assess the usability of the VMood app. Participants will complete the full suite of outcome measures at baseline (aside from the SUS), every three months until completing the intervention and at three months after completing the intervention (follow-up). The full suite of outcomes measures will be delivered online using REDCap electronic data capture tools hosted at the Institute of Population, Health and Development (PHAD) in Vietnam [44,45]. See Table 3 for a summary of the full suite of measures.

**Table 3:** Outcome measures.

Measure	Outcome	Frequency
***Clinical Outcomes–Depression/Anxiety***		
*PHQ-9	Depressive symptoms	Baseline, then monthly until 3 months post intervention
GAD-7	Anxiety symptoms	Baseline, then monthly until 3 months post intervention
***Clinical Outcomes–Quality of Life***		
WHOQOL-BREV	Functioning	Baseline, and at every 3 months until 3 months post-intervention
FAST	Alcohol use	Baseline, and at every 3 months until 3 months post-intervention
ASSIST–adapted	Tobacco use	Baseline, and at every 3 months until 3 months post-intervention
***Economic Outcomes–Cost Effectiveness Analysis***		
EQ-5D-5L	Functioning; can use this to calculate QALYs	Baseline, and at every 3 months until 3 months post-intervention
HEA–adapted	Costs attributable to health resource utilization	Baseline, and at every 3 months until 3 months post-intervention
WPAID:D	Indirect costs attributable to potential productivity gains	Baseline, and at every 3 months until 3 months post-intervention
***Usability Outcome***		
SUS	System (VMood) usability	At 3 months, then every 3 months until 3 months post-intervention

*Primary outcome will be depression caseness (binary outcome) at 3 months. Other clinical (depression) outcome will include PHQ-9 change scores.

#### Data collection

**Fig 1:**
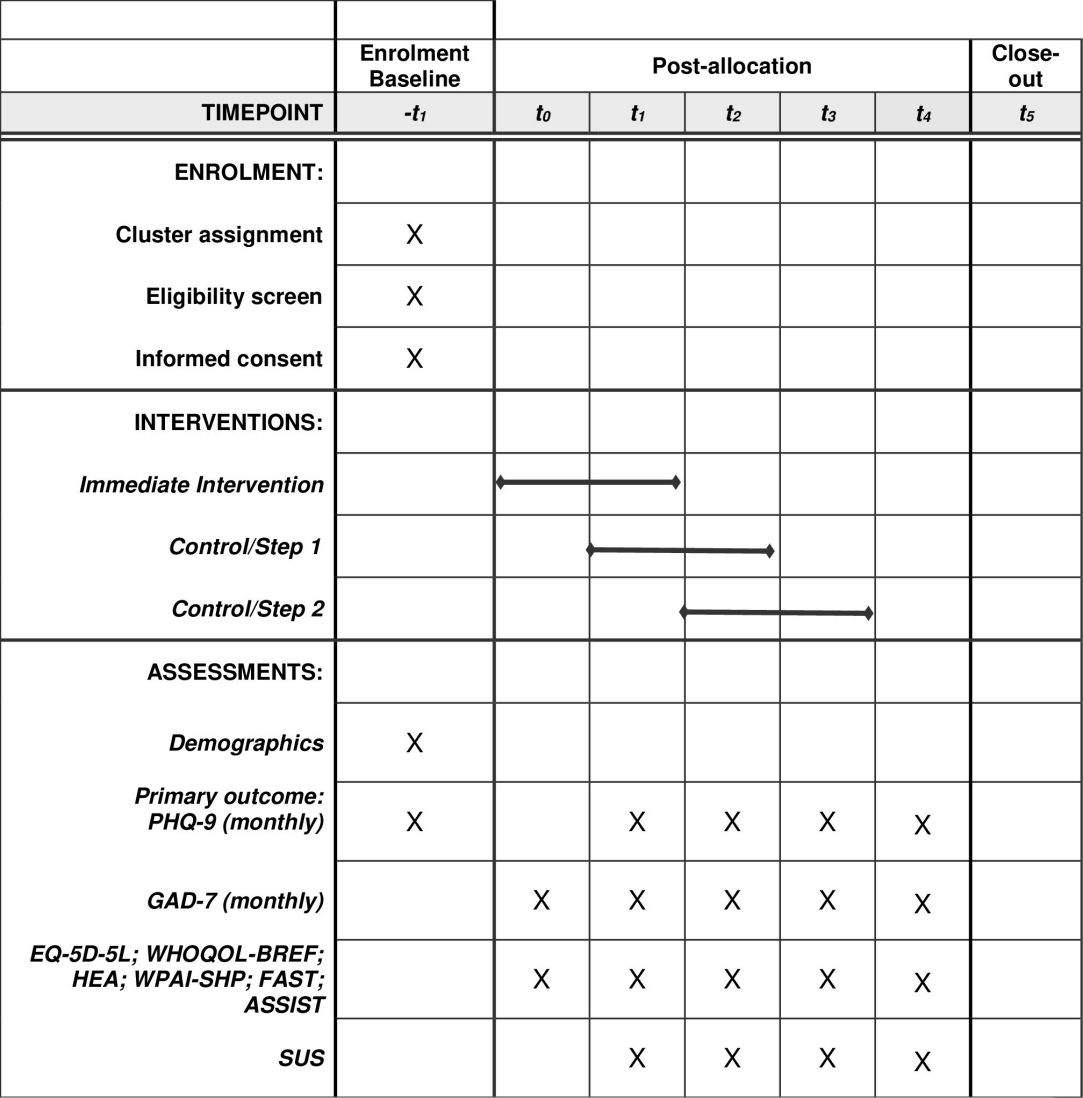
VMood schedule of enrolment, interventions, and assessments.

Participants will complete the full suite of outcome measures at baseline (aside from the SUS), every three months until completing the intervention and at three months after completing the intervention (follow-up). The full suite of outcomes measures will be delivered online using REDCap electronic data capture tools hosted at the Intitute of Population, Health and Development (PHAD) in Vietnam. Outcome measures will be disseminated using REDCap survey software [44,45] and will be completed by particpants at baseline and every three months, with a final assessment scheduled three months months after completion of the 3-month VMood intervention. See Fig 1 for full schedule.

### Sample size and power calculation

The power calculation assumes that each commune would recruit an average of 8 participants and retain 7 participants after attrition and that the proportion of depression caseness (defined as PHQ-9 score between 10–19) in the control group is 42%, the same as in the previous study [25]. The calculation shows that for a type I error rate of 0.05, and an intracluster correlation coefficient (ICC) of 0.01 (based on data from the previous study on the binary outcome of having depression), a total of 336 participants recruited from 48 clusters (i.e., communes) have at least 80% power to detect treatment differences when the proportion of depression caseness in the intervention group is ≤ 30% (Table 4 below) [46]. The power calculation was performed using Stata 17 [46].

**Table 4:** Power analysis.

Proportion of depression caseness in Intervention Condition	Power
0.30	81%
0.31	74%
0.32	66%
0.33	57%
0.34	48%

### Randomization and blinding

Randomization will be done using permuted blocks (to conceal allocation) and will be stratified by district. The randomization sequence will be developed and controlled by an individual not otherwise involved in the study to ensure fidelity and supervised by co-author HX. After baseline assessment, communes will be randomly assigned to the three sequences (Table 1) in a 1:1:1 allocation ratio stratified by district in variable block sizes implemented in SAS v9.4 to prevent anticipated allocation of the communes to the treatment sequences.

Full blinding of participants will not be possible due to the nature of the intervention.

### Statistical analysis

In preliminary analysis, the distributional aspects of the variables under consideration will be examined through descriptive statistics. These will include measures of location (mean, median, quartiles) and measures of dispersion (range, standard deviation, variance) for continuous variables, and frequencies of the levels of categorical variables. These measures will also assist with a data cleaning/screening procedure to eliminate any possible copying, handling, recording or measurement errors.

The general approach for analyzing all the types of outcomes arising from the study will be the Generalized Linear Mixed-effect Models (GLMM) for longitudinal and clustered measures, with the commune as the clustering variable. These models were used in the previous study [25] and can handle a wide range of types of outcomes including normal, binary, ordinal, and Poisson outcomes, and can account for the correlations for observations within the same clusters. Specifically, the primary endpoint (depression caseness based on PHQ-9) is a binary outcome and will be analyzed using random-effect logistic model, a special case of GLMM. The main independent variables in statistical model specifications will include indicator variables for visits (for secular trend), indicators for VMood intervention effects at months 3 and 6, and district (to control for trial initiation time) and baseline score as fixed effects. Additionally, these models included random effects for communes (the clusters) and for participants nested within communes to account for random variation between communes and between participants within the same commune. By properly setting up the GLMMs, a wide range of scientific hypotheses can be tested, including the primary ones regarding treatment effect estimation. The quality of statistical inferences will be substantiated through rigorous model checking and validation techniques. Data will be analyzed using the PROC MIXED, PROC GLIMMIX and/or PROC NLMIXED in SAS 9.4 software [47]. Subgroup analysis will be performed using the GLMM on the subsets of data formed by the subgroup.

When applying the above statistical models in practice, a potentially important issue is dealing with missing values, which is common in most studies. One advantage of employing the above GLMM is that it uses all the available data by including those study units with missing outcome values and produces valid inference under the assumption of missing at random, which is a more relaxed assumption of missing data mechanism than missing completely at random. The missing values will be regarded as missing at random when the nonresponse behavior is believed to be a function of observed data. The potential bias of these inferences will be quantified if these missing data were suspected to be nonrandom even after conditioning on the observed data using the selection models [48,49].

Another relevant issue is to properly account for treatment adherence (app usage) when evaluating the effect of VMood intervention. Two well-established and complementary evaluation methods will be used to evaluate the impact of the intervention accounting for treatment nonadherence: Intention-to-treat (ITT) and Complier Average Causal Effect (CACE). The primary approach is to apply the method of ITT to estimate causal effects of the assignment to treatment on the trial outcomes for all participants. The ITT has the merit of making use of randomization in RCT. Although ITT analysis is valid, one limitation of ITT analysis is that it estimates the program effectiveness rather than the method effectiveness. The program effectiveness depends on the proportion of compliers, which can change (e.g., when the public change their willingness to take the intervention after they learn the intervention works). Thus, CACE will be applied as a secondary analysis. The CACE analysis estimates causal effects of the assignment to treatment on the trial outcomes for the subgroup of compliers [47,50]. Thus, CACE analysis will complement the above ITT analysis to paint a more complete picture about the impact of treatment on groups of populations that would be of interest to individual patients.

### Economic evaluation

The economic analysis will determine the cost-effectiveness of the Vmood app intervention compared to the control condition (enhanced treatment as usual). Following best-practice recommendations of the Second Panel on Cost Effectiveness in Health and Medicine [51] and for the conduct of cost-effectiveness analysis alongside RCTs [52,53], (i) a trial-based cost-effectiveness analysis (CEA), generated exclusively with trial data; and (ii) a probabilistic model-based CEA, required to capture both the long-term costs and benefits of the intervention will be executed.

As per recommended guidelines for the conduct of CEA alongside RCTs, health resource use and health-related quality of life measures will be obtained directly from trial participants [52,53]. All costs will be estimated from trial data [40] including direct costs attributable to health resource utilization [40], intervention delivery and implementation [54], and indirect costs attributable to productivity impairment [41,42]. Costs will be reported by trial arm as cost per participant enrolled on an intent-to-treat basis. The number of QALYs accumulated during the study period will be estimated from patient reported outcomes collected from trial participants. Model development for capturing the long-term costs and benefits of the intervention will follow best practice recommendations for conceptualizing a model [55] and will be informed by systematic reviews [56] as well as recommendations for modelling the cost-effectiveness of interventions to treat depression [20].

Results will be reported as an incremental cost-effectiveness ratio (ICER) in US$/QALY and VND/QALY at prevailing exchange rates, both from a health-care sector perspective (which will exclude productivity costs) and from a societal perspective, using a 3% annual discount rate for costs and QALYs [51]. Cost-effectiveness will be evaluated at willingness-to-pay thresholds based on per capita gross domestic product (GDP), the approach recommended by the World Health Organization [57]. Uncertainty around ICERs will be quantified with 95% confidence intervals using non-parametric bootstrapping in the trial-based CEA, a method specific to applications with patient-level cost and health outcome data) [58] and cost-effectiveness acceptability curves. Finally, the reporting of the economic analysis will adhere to health economic evaluation reporting standards [59].

### Implementation

#### Data management

All outcome measure assessments will be administered through REDCap [44,45], a secure web application for survey administration and database management. The electronic assessment data will be stored on SFU’s cloud storage system. All app data will be stored on PHAD’s secure server. Access to these databases will be limited only to authorized team members (Co-PIs, PHAD Project Coordinator, SFU Research Manager). Non-SFU team members will be assigned an SFU computing ID through IT services for the purpose of accessing the SFU cloud storage system as required, or provided a secure URL to access the data. Hardcopies of printed transcripts and notes taken during data collection will be stored in a locked cabinet in a secured office. All data (excluding app data) will be stored on Canada’s Federated Research Data Repository (FRDR) [60] for future open access initiatives, as required by funders. Local copies of all data will be stored on the various servers and then destroyed after five years, according to Vietnamese data retention policy.

#### Confidentiality

We adhere to the best practice guidelines in app development. The VMood app will include use industry-standard end-to-end 256-bit encryption throughout the consent process to ensure privacy in the data collection, handling, and storage processes. Additional security measures will include two-factor authentication for participants to access their information. Study participants will be assigned a unique identification number. All data will be coded by that number and no identifying information will be used to ensure confidentiality. Should participants agree to be contacted for future studies during the consenting process, their names, along with corresponding identification numbers, will be stored on a password-protected encrypted file stored on SFU’s cloud storage system. Only authorized team members (Co-PIs, PHAD Project Coordinator, SFU Research Manager,) will have access to the file.

All app and other electronic data, including survey data gathered through REDCap, will be stored on PHAD’s secure server using industry-standard 256-bit encryption for protection of electronic health information. No identifiable information will be crossing borders. App data, stored on PHAD’s secure servers, will similarly be permanently deleted after five years according to Vietnamese data storage policy.

#### Retention strategies

Several strategies will be in place to promote retention. Participants in the intervention and control groups will receive a stipend of VND 100,000 (equivalent to CAD $5.00) after completing outcome assessments at baseline, and at every 3 months until 3 months post-intervention. Last, the app will include a progress bar that helps participants to track their progress in the app as a form of encouragement.

#### Fidelity testing and feasibility study

The overall research program aims to address the key knowledge gap on evidence supporting the use of digital health interventions by testing the effectiveness of the app-based VMood intervention in the Vietnamese community. This will be supported by initial feasibility testing including fidelity testing of the adaptation process from in-person to app format to ensure adherence to the original intervention and usability testing to assess the usability and acceptability of VMood, including participant recruitment, engagement, and retention rates. The fidelity testing will involve a convenience sample of participants from the previous trial. The usability testing will involve six communes in a different district from one of the eight participating provinces in Vietnam. Participants in the feasibility study will be included regardless of depression caseness, though we anticipate that individuals who download the app will likely have higher rates of depression-related symptoms compared to the general population. Results from the fidelity and usability testing will be published elsewhere once available.

## Discussion

This study aims to assess the effectiveness and cost-effectiveness of an app-based supported self-management intervention for depression (VMood) that has been adapted from a previously-tested, in-person intervention. VMood will be tested in eight provinces of Vietnam, where existing care for depression is extremely limited. The previous RCT of the in-person intervention demonstrated its effectiveness in reducing severity of depression among adults in community-based settings [56]. This program of research has consistently involved a strong partnership with MOLISA, with the intention of providing research evidence to inform the scale-up of depression care in Vietnamese communities. Following the original RCT, MOLISA expressed their interest in exploring digital mental health opportunities to promote a more cost-effective and feasible scale-up of the SSM model. This study will therefore assess the effectiveness of the VMood intervention for reducing the severity of depression compared with the in-person version of the intervention. It will also contribute cost-effectiveness evidence to inform the potential scale-up of the intervention throughout Vietnam should study results demonstrate intervention effectiveness.

The COVID-19 pandemic has had a substantial mental health impact worldwide, calling further attention to the urgency of increasing the availability of evidence-based mental health care [15]. The pandemic has also illuminated the inequities that exist related to mental health risk and access to care between countries and among populations within countries [61]. The rapid shift to the use of digital technologies in healthcare as a result of COVID-19 mitigation strategies early in the pandemic highlighted the potential of digital approaches to increase access to care, with widespread and growing support for the sustained use of these technologies [61] in addition to traditional in-person service delivery. In Vietnam, the government’s interest in shifting to an app-based delivery of SSM predated the pandemic, but the last three years have further underscored the potential for an app-based model to improve access to evidence-based care in a way that is both cost-effective and more feasible given the overwhelming resource constraints faced by the Vietnamese health and social services sectors.

This study builds on almost a decade of collaboration between the research team and MOLISA and other government stakeholders (e.g., MoH) in Vietnam. The Government of Vietnam has increasingly prioritized enhancing care for depression at the community-level in Vietnam, though availability of care for depression remains very low. This study has the potential to contribute substantially to the availability of care for mild to moderate depression in the country. Mobile phone coverage is high in Vietnam, with 131 cellular subscriptions per 100 people [62], meaning that a smartphone-based app will be widely accessible, including in regions where mental health care is limited or unavailable. App-based interventions can also help to promote help-seeking and access to care in the context of mental health stigma, which may discourage people from seeking care, and low mental health literacy [63].

One potential limitation of this study is that outcome measures were designed in Western contexts. To mitigate this, we ensured that the measures selected had either been validated in Vietnam or for the two measures that had not been (i.e., ASSIST and HEA), we worked closely with our Vietnamese partners to adapt the measures to enhance cultural validity. Further, most of the measures have been validated widely internationally, including for use digitally.

A second possible limitation is risk of attrition and bias. Control-group participants will be provided access to the VMood app with an introductory video on depression and asked to queue until they cross-over into the intervention group. During this period of waiting they may lose interest in the app or may seek alternative care. Despite this, we are confident most individuals will remain with the app as community-based mental health care in Vietnam is extremely limited. We will also ask participants to record any other care they receive during the control and intervention periods. In addition, to obtain a high follow-up rate for the 3-month intervention period, we will ensure sustained communication between social workers and participants via the app, and provide participants with appropriate incentives. For each assessment, to be conducted at baseline, and at three and six months, we will provide participants with a payment of VND 100,000 (equivalent of CAD $5).

A final possible study limitation is that it takes place in eight of Vietnam’s 63 provinces. Vietnam is a very diverse country, home to 54 ethnic groups, of which 53 are ethnic minority populations [64]. There is also considerable variation in language, culture, and customs between the country’s northern, central, and southern provinces. In recognition of this, our team, in close collaboration with MOLISA, carefully selected provinces across all three regions, with special attention given to the selection of districts and communes to ensure diversity in aspects such as population composition, economic status, and health services resource availability to achieve broad generalizability.

This study is part of a highly promising global movement to leverage digital technology to bring effective and low-barrier mental health care to people who would otherwise have little or no access to care. If the digital VMood intervention is found to be effective, research findings can contribute critical evidence to the emerging field of digital mental health by demonstrating that a low-barrier and low-cost intervention for depression can help begin to alleviate the global mental health crisis, especially in severely resource-constrained contexts where most of the world’s population resides.

## Supporting information

S1 Table SPIRIT 2013 checklist: Recommended items to address in a clinical trial protocol and related documents*. (DOC)

S1 File Hemming et al., 2018, Reporting of Stepped Wedge Cluster Randomised Trials: Extension of the CONSORT 2010 statement with explanation and elaboration. (PDF)

S2 File VMood study protocol. (PDF)
