# Supplementary material for: Evaluating the effectiveness and cost-effectiveness of a digital, app-based intervention for depression (VMood) in community-based settings in Vietnam: Protocol for a stepped-wedge randomized controlled trial
Source: PLoS One. 2023 Sep 5;18(9):e0290328. doi: 10.1371/journal.pone.0290328 (PMC10479903; doi:10.1371/journal.pone.0290328)
Supplement: S2 File — (PDF) [file pone.0290328.s003.pdf]

Study Summary

5.1.A. Provide a brief statement about the project written in lay language. Do not exceed 100 words and do not cut and paste directly from the study proposal.

The goal of this study is to conduct preliminary feasibility testing (fidelity and usability), along with pilot and test VMood, a Smartphone-based depression intervention that was adapted from an in-person supported self-management (SSM) intervention, in Vietnam. The SSM intervention was originally developed and tested in Canada. In previous work, our team demonstrated that the in-person SSM intervention was effective in reducing symptoms of depression among adults in Vietnam. Interviews with stakeholders in Vietnam showed the impact of COVID-19 on mental health and in-person health services and a growing need for innovative mobile health (mHealth) services. This study will help to establish a feasible, cost-effective, and scalable blended SSM intervention for adults with depression in Vietnam.

* **5.1.B.** Summarize the research proposal, including study purpose, hypothesis, study population, and research method.

This study will examine whether a supported self-management (SSM) intervention for depression can be adapted for delivery through a Smartphone app (VMood) in Vietnam. This ethics application covers the third and fourth phases of a series of projects funded by Grand Challenges Canada (GCC) and the Canadian Institutes of Health Research (CIHR) to implement and scale up a community-based intervention for managing depression.

- Phase 1: The GCC-funded project (MAC-FI) was a randomized controlled trial (RCT) that demonstrated the effectiveness of a SSM intervention utilizing task-sharing and a patient skills workbook called the Antidepressant Skills Workbook (ASW). Task-sharing involves the delivery of mental health services by non-specialist providers.

- Phase 2: The CIHR-funded implementation project (IRIS-DSV) helped to identify the drivers and barriers influencing the effective implementation of the SSM intervention in community-based settings in Vietnam, and enabled the team to work with government stakeholders to use the findings to support scale-up of the intervention across the country, now through a digital platform.

Results from Phase 2: IRIS-DSV suggest a mobile health (mHealth) adaptation of the original SSM intervention may help facilitate accessibility, scale-up and sustainability. This adaptation has taken on even greater urgency with the impact of COVID-19. This study (Phase 3) will also support a longer-term research plan to implement a “blended” model of community-based depression care in Vietnam that integrates both the original Phase 1: MAC-FI in-person model where appropriate, with an mHealth adaptation to ensure services reach all sectors of the country and population in a cost effective and equitable way.

The goal of this combined feasibility (fidelity and usability/acceptability testing), pilot, and community-based stepped-wedge cluster randomized implementation study is to test the implementation of an mHealth digital intervention adapted for use through a Smartphone app

(VMood) for depression. The specific study objectives of this randomized pilot feasibility study are to:

1. Establish implementation fidelity – in this context the degree to which the new mHealth intervention (VMood) is consistent with the conceptual and practical aspects of the in-person intervention;
2. Determine the acceptability/usability and feasibility of implementing VMood as a digital intervention to promote sustained user engagement for depression management in Vietnam,
3. Determine the potential effect of VMood on depression symptoms, and compare it with the results from the MAC-FI RCT.

Note the feasibility testing addition is PhD Candidate Chau's proposed dissertation work. The Phase 1: MAC-FI study demonstrated that one social worker will be able to manage approximately 1-2 chats at the same time, up to a maximum of 7.

For this Phase
We will engage the appropriate number of social workers depending on participant enrolment at a given time. Participants can contact their assigned social worker through a chat function or schedule an appointment to chat through the app to allow optimal utilization of social workers' time. Working remotely, the social worker will provide ongoing weekly support for using the skills in the ASW and monitor the participant's progress on acquisition and use of the skills. The app will also provide prompts to encourage participants to chat with their social worker when needed, or visit their local commune health centre if their condition worsens. To ensure continuity of care, each participant will be assigned one social worker who will support them over the course of the intervention. Training for social workers will be provided by staff in PHAD familiar with SSM and ASW who provided training to social workers and collaborators in the Phase 1: MAC-FI RCT. A full day orientation and training session on the project objectives, PHQ-9/GAD-7 and other relevant measures, ASW, and providing support through the app will be scheduled. The training video and online course on depression and how to use the apps will be developed and made available, with added mHealth components. Providing support services via chats will comprise a part of social workers' regular job responsibilities.

VMood app development: The app is being developed based on an extensive literature review conducted to establish best practices and ensure intervention fidelity adapting in-person community-based interventions to mHealth. Working with an experienced local app development company (DEHA Solution JSC, https://deha-soft.com/), the process is led by PHAD, our local partner on the SSM studies. Using an iterative design, the VMood prototype will be further developed entirely in Vietnamese before translation to English in consultation with a Participant Advisory Committee made up of participant volunteers who participated in the Phase 1: MAC-FI RCT. The Participant Advisory Committee will be asked to assess the cultural fidelity of the transition of activities from the in-person SSM intervention to VMood and will meet quarterly with the local study Investigators and Coordinator to review VMood app development/refinement and study progress.

<u>A. Feasibility Study</u>

The feasibility study will involve fidelity and usability and acceptability testing.

Fidelity Testing: this phase of the feasibility testing will investigate key elements of fidelity when adapting an in-person intervention to a digital format. Implementation fidelity is the degree to which an intervention is delivered as intended. Fidelity testing will involve three components described below. Additional details are provided in Section 5.6. Summary of Procedures.

Phase 1: Conceptualizing Fidelity: An embedded ethnography method will be used for this component, where participation as an "embedded researcher" in discussions around fidelity will provide insight into and help to inform its conceptualization within the context of adapting interventions from an in-person to a digital format. The process involved first defining exactly what fidelity means in this specific context, which included a review of the literature on both fidelity in general and fidelity in this context [see Chau PhD proposal]. The results from this review were provided in the background section. The tension between fidelity (usually advocated for by developers) and adaptation (usually advocated for by implementers) will serve as the analytical framework for conceptualizing fidelity. Next, discussions around fidelity amongst team members will take place through research team meetings (in-person and on Zoom) and personal communications (emails). A main source of data arose from the in-person team meetings held in Hanoi, Vietnam from August 22-25, 2022, where I obtained consent from team members to record their discussions and use them in my analysis.

Throughout, I will take detailed field notes summarizing and interpreting the discussions have been recorded. All discourse data will be transcribed. Key findings advocated for along the fidelity-adaptation continuum will be identified from these discussions.

Phase 2: Measuring Fidelity will involve the app being tested by participants (n=10) and providers (social workers/collaborators; n=10) who have prior experience with the in-person SSM intervention for a 2-week period, followed by interviews with the participants and providers (n=20). Experts familiar with CBT principles (n=6) will be recruited in Canada (n=3) and in Vietnam (n=3) to provide feedback on the conceptual components of the intervention adaptation.

App usage data will track the amount of program content received by participants. This will be captured using the proportion of participants who complete ≥50% of core app components and access ≥1 coaching sessions(s) (<u>D2: dose</u>). App usage data will also capture the number of times participants log in and engage with the app, in both duration and intensity. Note that app usage data will be captured merely to inform the qualitative methods in Phase 3: Enhancing Fidelity.

Phase 3: Enhancing Fidelity will be explored through exit interviews with the experts (n=6), participants (n=10), and providers (n=10) to gain insights into quality of delivery (D3) and participant responsiveness (D4), and identify necessary adaptations (D5).

Data Analysis: <u>Discourse data</u> will be analyzed using thematic content analysis as outlined by Braun & Clarke to identify and analyze common themes across transcripts that capture important meanings and patterns in the data. Thematic analysis is the most commonly used analysis method in qualitative research. A coding framework will be developed deductively, informed by

the fidelity-adaptation analytical framework, and inductively through analysis of the qualitative data. App usage data will be analyzed using basic descriptive statistics to capture the fidelity dimension dose (D2) and their main purpose will be to inform the qualitative interviews. Interview data will be analyzed using thematic content analysis, similar to discourse analysis. The coding framework will be developed deductively, informed by the core dimensions of the implementation fidelity framework,[5] and inductively through analysis of the qualitative data.

Data triangulation and reflexivity will be used to help ensure the trustworthiness of the data analysis and research findings. Triangulation will be used to explore congruence and divergence between data sources, thus increasing the rigor of the findings and reflexivity aided by the detailed field notes and memos made during the qualitative coding process will help ensure trustworthiness of the research findings. These strategies will provide an audit trail of the analytic process to ensure that other researchers can arrive at a comparable conclusion following the same processes. The audit trail will also include detailed records of the study's methods and procedures. NVivo software will be used for all qualitative analysis.

Usability and acceptability testing: this phase of the feasibility testing will involve assessing the usability and acceptability of VMood as a digital intervention to promote sustained user engagement. The final prototype will be deployed for a 3-month period to convenience samples in four communes from one district for usability testing. Communes will be purposely selected, in partnership with MOLISA, for diversity in population composition (i.e., men/women; elderly/young) and economic status (i.e., urban/rural; poorer/richer). Usability testing will utilize a mixed methods sequential explanatory design, with quantitative data collected first through the app testing phase and qualitative data collected subsequently with select participants to provide clarity and in-depth understanding around the quantitative data.

Samples will consist of app users who self-identify as experiencing symptoms of depression (n=30) and providers (n=10) new to the intervention. Recruitment for users will take place over a one-month period and the final sample size will be dependent on the number recruited and could be higher than the target 30. If and once the 30 threshold is reached, purposive sampling will be used to recruit additional participants for gender, urban/rural, and elderly/young.

App users will download *VMood* and be asked to complete the registration and consent forms. Once they provide consent, they will be asked to complete the depression screening measure, and be assigned a social worker to deliver virtual support. Depression screening will involve the Patient Health Questionnaire (PHQ-9). The PHQ-9 allows us to measure changes in depression symptom severity and has been validated for use with mHealth apps. Where participants exhibit severe mental illness, including the presence of suicidal ideation as measured on the PHQ-9 (question #9: Thoughts that you would be better off dead, or of hurting yourself in some way.), they will receive immediate information on how to access the nearest psychiatric hospital or outpatient clinic through the app. They will be asked for their consent for the app to send a notification to their CHC. A CHC staff member will then connect to assess whether they need to be referred to a tertiary psychiatric facility or other service. Any social workers or other staff who may be negatively affected as a result of participation in the study will be offered appropriate and timely support.

Quantitative Indicators: Key demographic information, such as age, sex, gender identity, education level, marital status, ethnicity, migrant status, occupation, and prior hospitalization due to mental illness, will be captured for both app users and social workers at baseline (0 months) through the app.

An exit survey will be administered to both app users and social workers upon intervention completion (3 months) to examine acceptability (Client Satisfaction Questionnaire) and overall feasibility (System Usability Scale). The Client Satisfaction Questionnaire (CSQ-8) is an 8-item Likert-type scale with four response options for measuring client satisfaction with services. The SUS is an industry standard, validated 10-item Likert-type scale with five response options for measuring usability with 0 (strongly disagree) to 4 (strongly agree). SUS has been validated for use across a number of digital technologies and is designed to support comparison across products. Participants will be asked to complete this embedded survey upon completion of the usability testing (3 months).

App usage data using built-in analytics will include metrics for evidence on implementation outcome adoption (e.g., rates of enrolment, rates of attrition, use and engagement, responsiveness to text messages and video chat requests from social workers).

Qualitative Methods:
Interviews*:* Semi-structured interviews will be conducted with app users identified as high (n=10) and low (n=10) completers of the intervention as well as the social workers (n=10) to provide clarity on the quantitative indicators and explore in-depth all implementation outcomes. Additional interviews will be conducted until saturation in key themes as identified in the implementation outcomes framework is reached. See **Attachments** for draft interview guides for app users and providers. Interview guides may be modified based on responses to the quantitative indicators.

Focus Groups: Focus groups (n=3) will be conducted to review the functionality of the VMood app, one with each of the three groups recruited for the interviews (high app user completers, low app user completers, providers), with six participants each. Participants will be selected from the groups who participated in the interviews using purposive sampling for socio-demographic variables (e.g., age, gender, urban vs rural). Focus groups will examine participant perspectives on VMood usability, including experience with digital interventions and cultural validity of VMood to inform implementation outcomes acceptability, appropriateness, and feasibility. Focus groups will take about 60-75 minutes each. See **Attachments** for the draft discussion guides for app users and providers. Focus group discussion guides may be modified based on responses to the quantitative indicators.

B. Pilot Study

The pilot intervention will involve participants from a province who are not familiar with SSM. The province will be determined in collaboration with our partner MOLISA (Ministry of Labour, Invalids and Social Affairs, Vietnam). Four rural communes in the province with similar sociodemographic characteristics will be randomly selected using a list provided by MOLISA.

These four communes will then be randomly assigned to intervention and comparison communes. Participants in the intervention communes will receive VMood for three months.

Those who agree will be invited to download the app into their mobile phone and begin the enrollment process. Consent to participate in the program will be first obtained through the app. Participants will be made aware of any potential security and privacy risks associated with using the app, including the most common issue arising from the loss of the device, and be provided with the necessary security measures to minimize the risks should this occur. All those who consent will then be allowed to open the PHQ-9/GAD-7 screening module in the app. The PHQ-9 will also be tested to determine if it can provide feedback in the app to users on changes in the severity of their illness.

We aim to recruit at least 100 participants (50 in each commune cluster) as this is a typical sample size for a feasibility pilot study of this kind.

Intervention communes: If participants meet caseness criteria on the PHQ-9/GAD-7 measure (score $\geq$ 10), users will be enrolled in the study and will receive the rest of the intervention through the app supported by video instructions on applying the skills in the SSM workbook. The PHQ-9/GAD-7 will be completed monthly (enrollment, month 1, month 2, month 3), with a follow-up at month 6.

Comparison communes: If participants meet caseness criteria on the PHQ-9/GAD-7 measure (score $\geq$ 10), users will be enrolled in the study and receive an information PDF file and a short video describing depression and existing services available. The PHQ-9/GAD-7 will be completed monthly (enrollment, month 1, month 2, month 3), with a follow-up at month 6.

Follow-up will be done by telephone to help capture dropouts and those who never use the app. Where participants exhibit severe mental illness, including the presence of suicidal ideation as measured on the PHQ-9 (question #9: Thoughts that you would be better off dead, or of hurting yourself in some way.), they will receive immediate information on how to access the nearest psychiatric hospital or outpatient clinic through the app. They will be asked for their consent for the app to send a notification to their CHC. A CHC staff member will then connect to assess whether they need to be referred to a tertiary psychiatric facility or other service. Any social workers or other staff who may be negatively affected as a result of participation in the study will be offered appropriate and timely support.

Pre-and post-intervention surveys: A survey has been developed capturing the intended secondary outcome measures of the subsequent randomized implementation study and include the effect of the intervention on quality of life and overall health and economic status (WHOQOL-BREV), along with use of alcohol and tobacco products (FAST, ASSIST-adapted). The subsequent randomized implementation study will involve a cost-effectiveness analysis to capture VMood's costs and benefits. Corresponding measures estimating health-related quality of life (EQ-5D-5L), a costs attributable to health resource utilization (HEA-adapted for the Vietnamese context), and indirect costs associated with productivity gains (WPAI:SHP) will be included. Lastly, two wrap-up questions (1. How long did it take to fill this questionnaire? And 2. Do you have any comments about the questionnaire?) will help capture the burden on

participants and their initial thoughts. This full suite of measures will be administered at baseline and at 3 months [administration method TBD] to provide information on feasibility and acceptability in preparation for the implementation study by reviewing length for completion and any comments. Note the 3 months survey will also include the System Usability Scale (SUS). See **Attachments** for the survey. This survey will be translated to Vietnamese once we are closer to launching the pilot study. We will ensure Vietnamese/English language semantic equivalence through forward-backward translation.

Semi-structured interviews: (N=20) will be conducted with a random sub-sample of the participants from the intervention communes to ensure intervention fidelity (e.g., adherence to the skills in the ASW, completion of the key components of the app), usability (e.g., "I thought the system [app] was easy to use"), acceptability (e.g., reactions to and willingness to use mHealth approaches for mental health), and cultural validity (e.g., social and cultural factors affecting phone usage and the ability of health workers to promote mHealth). We will invite **social workers** (n=10) and **participants** (n=10) involved in the pilot intervention to participate in these qualitative semi-structured interviews. Interviews with both stakeholder groups will include questions for gender analysis, including questions such as (for social workers) "To what extent are female social workers expected to provide more emotional support than male social workers?" and, (for participants) "What kind of family support do you receive from your spouse?" Analysis will include an examination of gender-based differences in experience and outcomes with using the app.

Interviews will be conducted by a co-applicant (or grad student), with the aid of a local interpreter. We will prepare the interview guide in consultation with the Participant Advisory Committee, and pre-test the interview guides with PHAD Vietnamese staff not associated with this project. All interviews will be audio recorded, transcribed and translated to English. Interview notes will also be documented to describe non-verbal behaviour, impressions, content of the interview, and influence of the interviewer on data collection. Please note interview guides for this pilot study will be submitted at a later date in an amendment, once we are closer to data collection.

C. Implementation and Scale-up Study

The Implementation and Scale-up study (funded by GCC as the "AIMDIV Project" and attached here) will involve the same components as the pilot study, but expanded to an additional 8 provinces in Vietnam through a two-armed stepped-wedge cluster randomized implementation study. The target number of participants is 786 for the overall implementation study, and 60 for the interviews. As part of the implementation and scale-up project, we plan to pilot and test the VMood intervention with additional individuals, including populations in different geographic and socioeconomic circumstances (e.g., migrants in industrial zones), which will be identified in collaboration with the Project Advisory Committee as the study progresses.

Our team has finalized the suite of measures to be administered to users through the app, as detailed earlier in the pilot study section. Please note interview guides for this implementation study will be submitted at a later date in an amendment, once we are closer to data collection.

The Implementation and Scale-up amendment also includes an economic evaluation: We intend to execute (i) a trial-based cost-effectiveness analysis (CEA), generated exclusively with trial data; and (ii) a probabilistic model-based CEA, required to capture both the long-term costs and benefits of the intervention (typically conducted over 5-year, 10-year,and lifetime horizons). As per recommended guidelines for the conduct of CEA as part of randomized implementation studies, we will obtain health resource use and health-related quality of life measures directly from study participants. The use of model-based economic evaluation can provide context- and setting-specific evidence of the long-term costs and benefits attributable to competing treatment strategies. Evidence from model-based CEA can thus provide the economic argument to support the scale up of effective treatment for people living with CMD in Vietnam (and in member countries of the Association of Southeast Asian Countries facing similar mental health challenges, capacities, and lost productivity from untreated depression. Model development will follow best practice recommendations for conceptualizing a model and will be informed by systematic reviews as well as recommendations for modelling the cost-effectiveness of interventions to treat depression.

Study Population for the Pilot and Implementation Studies:
Scale-up of the VMood intervention will target two populations: 1) adults living with depression, and 2) service providers (social workers who will assist with delivering the intervention and primary health care workers who will assist with app user participant recruitment). Most of the service providers will be receiving evidence-based mental health training for the first time as part of their professional skill set. While the ultimate target population is people living with depression (and anxiety) and their families, the health workers and community-based care providers are integral to the success of the innovation and will also benefit from its implementation. An additional anticipated benefit of the innovation will be to help reduce the widespread stigma, fear, and discrimination experienced by individuals with mental health problems.We will be conducting the VMood and AIMDiV projects in conjunction with each other, with preliminary results from VMood helping to inform the AIMDiV project set up and data collection.

A Project Advisory Committee will be organized in the first year of the project to provide oversight on implementation. Membership will include senior representatives from relevant Ministries and other stakeholder groups (i.e., Universities). It will be chaired by Mr. Hoi (Project Co-Leader and Vice Minister, MOLISA) and Professor Harry Minas (representing the Project Team). Quarterly meetings of the Project Advisory Committee throughout the four years of the project with members of the project team will ensure coordination of project activities and timely and focused feedback to the responsible Ministries for scale-up and sustainability.

Please note that the AIMDiV proposal (attached in section 9) includes activities that will be undertaken by the Government of Vietnam that are not research activities requiring ethics review, namely the piloting and implementation of an Improved Mental Health Information System (IMHIS) with the Ministry of Labor, Invalids and Social Affairs in Vietnam and the training program for MOLISA staff to prepare them for AIMDiV scale-up. These activities will be overseen by MOLISA. The entirety of the AIMDiV proposal is attached to this amendment for reference.

Data Analysis for the Pilot and Implementation Studies
Primary analysis will be a thematic analysis of the qualitative interview and focus group data as outlined by Braun & Clarke to identify and analyze common themes, which capture important meanings and patterns in the data. Coding will be carried out on all data by three members of our team (one co-applicant and two research assistants). We will apply a gender-based analysis by incorporating gender within the framework. Data triangulation and reflexivity will be employed to help ensure the trustworthiness of the data analysis and research findings. NVivo software will be used for all qualitative analysis.

Analysis will also involve a calculation of the proportion of participants who complete the core components (primary outcome), as indicated by completion of >90% of the skills in the VMood app and chatting with their assigned social worker (text or voice) at least once during the three-month treatment.

Secondary analysis will involve Generalized Linear Mixed-effects Models (GLMMs) to analyze changes in the pre- and post-surveys to provide quantitative evidence of the impacts of the VMood intervention, including intervention fidelity, usability, and acceptability. GLMMs are the recommended statistical methods when analyzing clustered and longitudinal data as they use all available data, allow for greater flexibility when modeling time effects, and are able to more appropriately handle missing data at random compared with other widely used methods for the analysis of longitudinal data (e.g., repeated-measures analysis of variance) We will compare results with those from the Phase 1: MAC-FI RCT.

Dissemination Plan for Results: We will use an integrated knowledge translation (iKT) approach with our government partner MOLISA, the Project and Participant Advisory Committees and other stakeholders. We will hold regular meetings between PHAD and MOLISA and the Committees, and an annual, in-person meeting with the study team, MOLISA, and others. In addition, we plan to disseminate study findings at global mental health (GMH) and implementation science conferences, both locally and internationally, and through peer-reviewed publications in high impact journals.

5.2. Inclusion Criteria
Participant Advisory Committee: Participant volunteers who previously participated in the in-person SSM intervention as part of the MAC-FI RCT.

Project Advisory Committee: senior representatives from relevant Ministries and other stakeholder groups (i.e., Universities) from Vietnam.

Fidelity Testing:
Experts**:** Experts familiar with CBT principles; 3 from Canada and 3 from Vietnam.
App Users**:** individuals who have prior experience with the in-person SSM intervention through the Phase 1: MAC-FI RCT.
Providers**:** social workers or social collaborators who have prior experience with the in-person SSM intervention through the MAC-FI RCT.

Usability, Pilot, and Implementation App User Participants: aged 18–60 years, currently living in the participating intervention and comparison communes, have cellphones with internet access, and able to provide informed consent.

Usability, Pilot, and Implementation **Providers:** social workers who fall under MOLISA's jurisdiction who have received training on the SSM intervention from PHAD.

5.3. Exclusion Criteria

Fidelity Testing: individuals who did not participate in the Phase 1: MAC-FI RCT.

5.4. Recruitment

Provide a detailed description of the steps you will use to recruit participants. Include:
a) How will prospective participants be identified?
b) By what means will recruitment be done (e.g., public posting, direct contact, third party recruitment, etc.)?
c) Who will contact prospective participants?
d) If recruitment will occur in person, what sites will be used (e.g. doctor's office, hospital clinic, etc.)?
e) Attach all materials, including letters of initial contact, posters, scripts and advertisements, to Box 9.4.

We will recruit Project Advisory Committee members based on recommendations from our MOLISA partners and chair of the Project Advisory Committee, Dr. Minas, based on their field of expertise at the provincial and national levels. A member of the PHAD team will contact these participants using the Project Advisory Committee Recruitment Script by telephone or email, depending on preference of the member, inviting them to enroll and commit to participating in regular committee sessions throughout the study. Meetings will occur quarterly throughout the four years of the project, for a total of 16 meetings. Contact information will be obtained from public official information available through the respective organizations. We will obtain informed consent at the first in-person meeting.

For the Participant Advisory Committee and fidelity testing component, we will identify participants through a convenience sample using records from the IRIS-DSV project (REB # H20-02658), which is an expansion of the qualitative component of the MAC-FI study (SFU REB #2016s0095). In Phase 2: IRIS-DSV we recruited participants from the same communities as MAC-FI. Some of these participants had participated in the Phase 1: MAC-FI RCT. Participants from IRIS-DSV were asked to provide consent to be contacted in the future for participation in other related studies. For the Participant Advisory Committee and fidelity testing participants, we will identify participants through this list from IRIS-DSV. We will recruit the rest of the sample through open invitations to community members to contact their local commune health centres if they are interested. PHAD research personnel will contact potential participants by phone using the submitted recruitment script.

We will recruit app user participants for the usability/acceptability testing from four communes in one district. Communes will be purposely selected, in partnership with MOLISA, for diversity

in population composition (i.e., men/women; elderly/young) and economic status (i.e., urban/rural; poorer/richer). Recruitment will take place over a one-month period and the final sample size will be dependent on the number recruited and could be higher than the target 30. If and once the 30 threshold is reached, purposive sampling will be used to recruit additional participants for gender, urban/rural, and elderly/young.

We will recruit app user participants for the VMood pilot and implementation studies through local commune health centres located across the 9 provinces in Vietnam. Consultations will be held with our partner, the Ministry of Labor, Invalids and Social Affairs (MOLISA) of Vietnam to determine the participating provinces and communes in the first quarter of the project. Staff at local commune health centres will recruit potential VMood users through normal health communication (e.g., social media) and through various community engagement events. Commune health centre staff will explain the study further to interested participants. As the original SSM pilot and RCT resulted in recruitment of more women than men, we will use purposive sampling once we reach a threshold to attempt to recruit equal numbers of women and men. This will allow us to examine whether gender, a key social stratifier, affects acceptability and feasibility of implementing the mHealth app and the effect of VMood on depression symptoms. Staff at the local commune health centres, trained by PHAD staff on study objectives, will help publicize the study, facilitate recruitment, and direct interested individuals to the app.

Social worker providers, employed through MOLISA, will be recruited through official MOLISA channels of communication by MOLISA personnel.

We will invite social workers and app user participants involved in the pilot and implementation to participate in qualitative semi-structured interviews. We will use purposive sampling to recruit equal numbers of women and men for the interviews.

The national coordinator (based at MOLISA) who has been involved in recruitment throughout the program of research, will support recruitment for all aspects of the study and support liaison with local CHCs.

5.5. Use of Records

We will use previous records from the IRIS-DSV project (REB # H20-02658), which is an expansion of the qualitative component of the MAC-FI study (SFU REB #2016s0095) to invite participants to take part in the Participant Advisory Committee, fidelity testing, and the VMood intervention. In IRIS-DSV we recruited participants from the same communities as MAC-FI. Some of these participants had participated in the Phase 1: MAC-FI RCT. Participants from IRIS-DSV were asked to provide consent to be contacted in the future for participation in other related studies.

We will keep and refer to a password protected master list with participant and provider IDs and contact information collected in the Participant Advisory Committee, usability, pilot, and implementation stages of VMood to recruit for follow up interviews. This information will be stored for 7 years in an SFU approved cloud storage system, after which time it will be

deleted/destroyed, after which time it will be deleted/destroyed.

All app data will be stored on PHAD's secure data centre using industry-standard 256-bit end-to-end encryption and will be similarly destroyed after 7 years. All other data will be stored on Canada's Federated Research Data Repository (FRDR) for future open access initiatives, as required by funder GCC.

*** 5.6. Summary of Procedures**

Describe briefly in a step-by-step manner what the researcher will be doing with participants, after they have been recruited and consented.

Participant Advisory Committee members will be asked to assess the cultural fidelity of the transition of activities from the ASW to the VMood and will meet quarterly with the local study Investigators and Coordinator to review the VMood app development and study progress.

A. Feasibility Study

Fidelity Testing

Phase 1: Conceptualizing Fidelity: At the team meetings held in Hanoi, Vietnam, the team reviewed the core elements and discussed in-depth best ways to incorporate into the app. The team also identified some elements that could be adapted for an app format (D5: program differentiation). For any adaptive interventions, systematically measuring program differentiation is key to assessing how a fidelity-adaptation balance can be reached. For this adaptation to a digital format, the team agreed various changes could be made to suit the new modus to engage users (e.g., reminders, encouraging messages), which is one of the key considerations for this context. Additional discussions around fidelity amongst team members will take place through research team meetings (in-person and on Zoom) and personal communications (emails).

Phase 2: Measuring Fidelity will involve the app being tested by participants (n=10) and providers (social workers/collaborators; n=10) who have prior experience with the in-person SSM intervention for a 2-week period, followed by interviews with the participants and providers (n=20). Experts familiar with CBT principles (n=6) will be recruited in Canada (n=3) and in Vietnam (n=3) to provide feedback on the conceptual components of the intervention adaptation.

Experts will be invited to review the workbook and MAC-FI protocol for delivery of the in-person intervention. They will subsequently be asked to review the app in its entirety for fidelity comparisons with the in-person intervention. Informed by a typology of adaptations summarized by Perez et al., experts will be asked to log their thoughts on: 1) additions or new components; 2) deletions or radical modifications to the intervention components that it no longer resembles the original one; 3) minor or major modifications to an existing component (e.g., changes in the intensity of administration). These logs will help to systematically track participant perceptions on any adaptations made and their thoughts on the impact of these adaptions to the intervention (D5: program differentiation), which will be explored through interviews in Phase3 – Enhancing Fidelity. Additionally, experts in Vietnam will be asked questions assessing cultural fidelity of the app to ensure relevance with local context.

Participants and providers will be invited to engage with the app for two weeks (D1: adherence). Each participant will be assigned a unique provider. Participants will similarly be asked to log their thoughts in the same manner as instructed with the experts. Participants will be encouraged to engage with their assigned social worker through the app.

Phase 3: Enhancing Fidelity: will be explored through exit interviews with the experts (n=6), participants (n=10), and providers (n=10) to gain insights into quality of delivery (D3) and participant responsiveness (D4), and identify necessary adaptations (D5). Quality of program delivery (D3) questions will address the quality of interactions and the degree to which interactive activities focused attention on desired core elements of the app. Participant responsiveness (D4) questions will examine the extent to which participants felt they were engaged by and involved in the activities of the app along with their level of satisfaction with the intervention. Program differentiation (D5) questions will examine whether participants noticed any key changes in the digital format and their opinions on these changes (what they liked, did not like). Questions will also explore participant logs on program differentiation and the impact, if any, of any adaptations on the intervention. Specific questions will be informed by the App Adaptation Fidelity Checklist [see **Attachments**]. Also see **Attachments** for the interview guides with experts, app users, and providers. Interview guides may change based on app usage data and participant logs. Through an iterative process, the app will be modified based on participant feedback. Fidelity will be monitored closely so that all adaptations are documented and their possible impacts captured through usability testing.

Interview and Focus Group Administration: All interviews will be conducted in person, where possible (i.e., local COVID-19 pandemic and other health regulations permitting). Chau, the PhD candidate, will conduct all interviews, with a PHAD team member assisting with day-of interpretations. All current local safety protocols will be followed. All interviews will be recorded, transcribed, and translated to English using forward and backward translation. In addition, Chau will record detailed field notes during and after the interviews and to capture thoughts and observations about the interactions that could help inform the data analysis process.

Usability and Acceptability Testing

App users will download *VMood* and be asked to complete the registration and consent forms. Once they provide consent, they will be asked to complete the depression screening measure, and be assigned a social worker to deliver virtual support. Depression screening will involve the Patient Health Questionnaire (PHQ-9). The PHQ-9 allows us to measure changes in depression symptom severity and has been validated for use with mHealth apps. Interested participants will be included regardless of depression caseness, as measured by a score of >4; however, we anticipate individuals who participate and download the app will have higher rates of depression-related symptoms compared to the general population. We have also included the Generalized Anxiety Disorder (GAD-7) measure, a brief 7-item scale, as it is a validated measure of anxiety, which often co-occurs with depression.

The PHQ-9/GAD-7 measure (primary outcome measure for the subsequent randomized implementation study) will be embedded within the app and administered monthly. App users will be invited to test the app for a 3-month period. We will ask app users to utilize the built-in social support function, engaging the social workers, who will help guide app users through the

app and intervention components. Social workers will also be trained to check-in on the app users (e.g., when there is inactivity for 2 weeks) and provide supportive coaching as needed. Social workers will receive training on depression, addressing stigma, providing coaching via the app, and emergency and suicide management through an online training program developed by PHAD. MOLISA will direct staff to complete the course and provide time to do so. A certificate of completion will be provided.

Quantitative Indicators
Demographics will be captured for both app users and social workers. An exit survey will be administered to both app users and social workers upon intervention completion (3 months) to examine acceptability (Client Satisfaction Questionnaire) and overall feasibility (System Usability Scale).

App usage data using built-in analytics will include metrics for evidence on implementation outcome adoption (e.g., rates of enrolment, rates of attrition, use and engagement, responsiveness to text messages and video chat requests from social workers).

Qualitative Methods
Interviews*:* Semi-structured interviews will be conducted with app users identified as high (n=10) and low (n=10) completers of the intervention as well as the social workers (n=10) to provide clarity on the quantitative indicators and explore in-depth all implementation outcomes. Questions will explore implementation outcomes acceptability (e.g., App user: please describe the overall experience of using the VMood app; Provider: did you feel the training you received adequately prepared you for your role supporting app users through VMood?), appropriateness (e.g., App user: would you recommend the VMood app to a friend or family member experiencing symptoms of stress, worry, and depression?; Provider: do you think that the VMood app should be expanded, or scaled-up, across Vietnam?), and feasibility (e.g., App user: did you use the social worker support function in the app?; Provider: Approximately how many app users have you worked with through the VMood app?). Interviews will take about 30-60 minutes each.

Focus Groups: Focus groups (n=3) will be conducted to review the functionality of the VMood app, one with each of the three groups recruited for the interviews (high app user completers, low app user completers, providers), with six participants each. Participants will be selected from the groups who participated in the interviews using purposive sampling for socio-demographic variables (e.g., age, gender, urban vs rural). Focus groups will examine participant perspectives on VMood usability, including experience with digital interventions and cultural validity of VMood to inform implementation outcomes acceptability, appropriateness, and feasibility. Focus groups will take about 60-75 minutes each.

Interview and Focus Group Administration: I will conduct the first two interviews with members from each group and moderate all focus groups, with a PHAD team member assisting with day-of interpretations and who will also help to observe and monitor the interactions of the individuals in the focus groups (e.g., nonverbal cues, speaking order) for additional analysis insight. The PHAD team member will assist with completion of the remaining interviews. All interviews and focus groups will be recorded, transcribed, and translated to English using

forward backward translation. The PHAD team member and I will also record detailed field notes during and after the interviews and focus groups to capture thoughts and observations about the interactions that could help inform data analysis.

B. Pilot Study

Intervention communes: If participants meet caseness criteria on the PHQ-9/GAD-7 measure (score ≥ 10), users will be enrolled in the study and will receive the rest of the intervention through the app supported by video instructions on applying the skills in the SSM workbook. The PHQ-9/GAD-7 will be completed monthly (enrollment, month 1, month 2, month 3), with a follow-up at month 6.

Comparison communes: If participants meet caseness criteria on the PHQ-9/GAD-7 measure (score ≥ 10), users will be enrolled in the study and receive an information PDF file and a short video describing depression and existing services available. The PHQ-9/GAD-7 will be completed monthly (enrollment, month 1, month 2, month 3), with a follow-up at month 6.

Follow-up will be done by telephone to help capture dropouts and those who never use the app. Where participants exhibit severe mental illness, including the presence of suicidal ideation as measured either on the PHQ-9 (question #9: Thoughts that you would be better off dead, or of hurting yourself in some way.), they will receive immediate information on how to access the nearest psychiatric hospital or outpatient clinic through the app. They will be asked for their consent for the app to send a notification to their CHC. A CHC staff member will then connect to assess whether they need to be referred to a tertiary psychiatric facility or other service. Any social workers or other staff who may be negatively affected as a result of participation in the study will be offered appropriate and timely support.

Pre- and post-intervention surveys: A survey has been developed capturing the intended secondary outcome measures of the subsequent randomized implementation study and include the effect of the intervention on quality of life and overall health and economic status (WHOQOL-BREV), along with use of alcohol and tobacco products (FAST, ASSIST-adapted). The survey also includes cost-effectiveness analysis measures to capture VMood's costs and benefits (EQ-5D-5L, HEA-adapted for the Vietnamese context, WPAI:SHP). Lastly, two wrap-up questions (1. How long did it take to fill this questionnaire? And 2. Do you have any comments about the questionnaire?) are included to help capture the burden on participants and their initial thoughts. This full suite of measures will be administered at baseline and at 3 months [administration method TBD] to provide information on feasibility and acceptability in preparation for the implementation study by reviewing length for completion and any comments. Note the 3 months survey will also include the System Usability Scale (SUS). See **Attachments** for the survey.

Semi-structured interviews: (N=20) will be conducted with a random sub-sample of the participants from the intervention communes to ensure intervention fidelity (e.g., adherence to the skills in the ASW, completion of the key components of the app), usability (e.g., "I thought the system [app] was easy to use"), acceptability (e.g., reactions to and willingness to use

mHealth approaches for mental health), and cultural validity (e.g., social and cultural factors affecting phone usage and the ability of health workers to promote mHealth).

Local health guidelines permitting, these activities will take place in person. In-person activities will be conducted by a PHAD team member (e.g., Co-PI Dr. Nguyen Vu Cong, Project Coordinator). A COVID-19 Health Check will be used for all participants at the start of each intervention.

For the Participant Advisory Committee and focus groups, we will identify participants through a convenience sample using records from the Phase 2: IRIS-DSV project, which is an expansion of the Phase 1: MAC-FI study. Participants from Phase 2 were asked to provide consent to be contacted in the future for participation in other related studies. We will only be contacting those who provided consent. In Phase 2 we recruited participants from the same 8 provinces as Phase 1. The provinces were: Quang Ninh, Thai Nguyen, Thanh Hoa, Da Nang, Quang Nam, Khanh Hoa, Long An, and Ben Tre. The location of the Participant Advisory Committee meetings and focus groups will depend on where the participants are located.

The pilot intervention will involve participants from a province who are not familiar with the supported self-management intervention. The province will be determined in collaboration with our partner MOLISA. Four communes (two randomly assigned to intervention and two randomly assigned to control/comparison) in the province with similar sociodemographic characteristics will be randomly selected using a list provided by MOLISA.

C. Implementation and Scale-up Study

For the Implementation and Scale-up Study, we will expand the VMood intervention to an additional 8 provinces with a target of 786 participants overall, and 60 interview participants. Scale-up will occur beyond the 786 participants included in the study.

The full survey will be administered at baseline (without the SUS measure), 3 months (intervention completion) and 6 months (follow-up). See **Attachments** for full survey.

Local health guidelines permitting, research activities (i.e., focus groups, Committee meetings, semi-structured interviews) will take place in person. In-person activities will be conducted by a PHAD team member (e.g., Co-PI Dr. Nguyen Vu Cong, Project Coordinator). A COVID-19 Health Check will be used for all participants at the start of each intervention.

Each individual app user will have their own account that require them to input a password to login and access their information. Two factor authentication will be used. We have added some text to the consent forms asking participants not to share their login information, but ultimately, we cannot control whether they share the app with others.

All app data, encrypted end-to-end with industry standard 256-bit encryption, will be stored on PHAD's secure servers in Vietnam. No one outside the research team will have access to the data.
